# Analysis of Early-Life Growth and Age at Pubertal Onset in US Children

**DOI:** 10.1001/jamanetworkopen.2021.46873

**Published:** 2022-02-04

**Authors:** Izzuddin M. Aris, Wei Perng, Dana Dabelea, Jody M. Ganiban, Chang Liu, Kristine Marceau, Olivia C. Robertson, Christine W. Hockett, Nicole L. Mihalopoulos, Xiangrong Kong, Megan M. Herting, T. Michael O’Shea, Elizabeth T. Jensen, Marie-France Hivert, Emily Oken

**Affiliations:** 1 Division of Chronic Disease Research Across the Lifecourse, Department of Population Medicine, Harvard Medical School, Harvard Pilgrim Health Care Institute, Boston, Massachusetts; 2 Department of Epidemiology, Colorado School of Public Health, University of Colorado Anschutz Medical Campus, Aurora; 3 Lifecourse Epidemiology of Adiposity and Diabetes Center, University of Colorado Anschutz Medical Campus, Aurora; 4 Department of Pediatrics, University of Colorado Anschutz Medical Campus, Aurora; 5 Department of Psychological and Brain Sciences, George Washington University, Washington, DC; 6 Department of Human Development and Family Studies, Purdue University, West Lafayette, Indiana; 7 Avera Research Institute, Sioux Falls, South Dakota; 8 Department of Pediatrics, Sanford School of Medicine, University of South Dakota, Vermillion; 9 Department of Pediatrics, The University of Utah, Salt Lake City; 10 Wilmer Eye Institute, Johns Hopkins School of Medicine, Baltimore, Maryland; 11 Department of Biostatistics, Johns Hopkins Bloomberg School of Public Health, Baltimore, Maryland; 12 Department of Population and Public Health Sciences, University of Southern California, Los Angeles; 13 Division of Neonatal-Perinatal Medicine, Department of Pediatrics, University of North Carolina at Chapel Hill, Chapel Hill; 14 Department of Epidemiology and Prevention, Wake Forest School of Medicine, Winston-Salem, North Carolina; 15 Diabetes Unit, Massachusetts General Hospital, Boston

## Abstract

**Importance:**

Earlier pubertal onset may be associated with an increased risk of chronic diseases. However, the extent to which growth in the first 5 years of life—an important developmental life stage that lays the foundation for later health outcomes—is associated with pubertal onset remains understudied.

**Objective:**

To assess whether changes in weight, length or height, and body mass index (BMI, calculated as weight in kilograms divided by height in meters squared) during the first 5 years of life are associated with earlier pubertal onset.

**Design, Setting, and Participants:**

This cohort study used data from 36 cohorts participating in the Environmental Influences on Child Health Outcomes program from January 1, 1986, to December 31, 2015. Participant inclusion required at least 1 anthropometric measure in the first 5 years of life and at least 1 measure of pubertal onset. Data were analyzed from January 1 to June 30, 2021.

**Exposures:**

Standardized velocities of weight, length or height, and BMI gain in early infancy (0-0.5 years), late infancy (0.5-2 years), and early childhood (2-5 years).

**Main Outcomes and Measures:**

Markers of pubertal onset for boys and girls, including age at peak height velocity (APHV), time to puberty score greater than 1, time to Tanner pubic hair stage greater than 1, and time to menarche. Multivariable regression models were used to estimate mean differences in APHV by growth periods.

**Results:**

Of 7495 children included in the study, 3772 (50.3%) were girls, 4505 (60.1%) were White individuals, and 6307 (84.1%) were born during or after the year 2000. Girls had a younger APHV (10.8 vs 12.9 years) than boys. In boys, faster weight gain (per 1-SD increase) in early infancy (β, −0.08 years; 95% CI, −0.10 to −0.06), late infancy (β, −0.10 years; 95% CI, −0.12 to −0.08), and early childhood (β, −0.07 years; 95% CI, −0.08 to −0.05) was associated with younger APHV after adjusting for the child’s birth year, race, and Hispanic ethnicity as well as maternal age at delivery; educational level during pregnancy; annual household income during pregnancy; prenatal cigarette smoking; whether the mother was nulliparous; whether the mother had gestational diabetes, hypertension, or preeclampsia; mode of delivery; prepregnancy BMI; gestational weight gain; and gestational age at delivery. Similar associations were observed for length or height and BMI gains during the same age periods. In girls, faster gains (per 1-SD increase) in weight (β, −0.03 years; 95% CI, −0.05 to −0.01) and height (β, −0.02 years; 95% CI, −0.04 to 0.00) in early childhood were associated with younger APHV. Faster BMI gain in late infancy was associated with earlier time to menarche, whereas faster BMI gain in early childhood was associated with earlier time to Tanner pubic hair stage greater than 1.

**Conclusions and Relevance:**

This cohort study found that faster gains in weight, length or height, or BMI in early life were associated with earlier pubertal onset. The results suggest that children who experience faster early growth should be monitored closely for earlier onset of puberty and referred as appropriate for supportive services.

## Key Points

**Question:**

Is early-life growth associated with age at pubertal onset?

**Findings:**

In this cohort study of 7495 children, faster gains in weight, length or height, or body mass index in early life in boys were associated with earlier pubertal onset (younger age at peak height velocity). In girls, faster gains in weight and body mass index were associated with earlier pubertal onset (younger age at peak height velocity and earlier time to menarche and Tanner pubic hair stage greater than 1).

**Meaning:**

Results of this study suggest that children with faster growth in early life may need to be monitored closely for earlier onset of puberty.

## Teaser

This cohort study examines whether there is an association between faster growth during a child’s early life and earlier pubertal onset.

## Introduction

Puberty is a key physiological milestone, characterized by maturation of the reproductive, musculoskeletal, neurodevelopmental, and cardiometabolic systems via distinct processes in boys and girls.^1^ During the past few decades, substantial concern has been raised about children entering puberty at younger ages in the United States and other countries.^2,3,4^ These observations have important clinical implications for children and their families that may necessitate counseling and intervention.^5^ Furthermore, children with earlier pubertal onset not only may be at increased risk for long-term chronic diseases^6^ but also may experience adverse consequences during adolescence, including psychosocial difficulties and dysmetabolism.^7^ A clearer understanding of the early life factors associated with pubertal onset is therefore important to develop intervention strategies to prevent earlier pubertal onset.

Existing evidence has pointed to an association between secular increases in prepubertal body mass index (BMI, calculated as weight in kilograms divided by height in meters squared) and timing of puberty,^8^ with higher BMI in childhood associated with earlier pubertal onset (eg, earlier age at menarche or more advanced pubic hair development).^9^ Other studies have shown that altered growth patterns during childhood may be associated with pubertal onset.^10,11^ Although the biological mechanisms have yet to be fully elucidated, faster growth could alter the early hormonal milieu and program the age at onset and rate of progression through puberty.^12^ Early growth may also be a marker of upstream exposures, such as environmental chemicals or excess nutrition, that are also associated with earlier pubertal onset.^13,14^ To date, the extent to which growth in the first 5 years of life—an important developmental life stage that lays the foundation for long-term health outcomes^15^—is associated with pubertal onset remains understudied. Few prospective studies have yielded data on growth measures across distinct age periods in the first few years of life (eg, early infancy, late infancy, early childhood), and even fewer have ascertained sensitive age periods during which early-life growth may be associated with pubertal onset. Furthermore, previous research on pubertal onset has been limited by small sample sizes^16^ and inclusion of only girls or homogeneous populations^17,18^ and often examined only 1 marker of pubertal timing (ie, age at menarche^19^).

To address the knowledge gaps, we leveraged existing data from cohorts participating in the Environmental Influences on Child Health Outcomes (ECHO) program, which has collected measures of pubertal onset and child growth across distinct life-course periods in a racially and geographically diverse population.^20^ We hypothesized that faster growth during the first 5 years of life would be associated with earlier pubertal onset in both boys and girls and that the association would be stronger during early childhood than during early or late infancy.

## Methods

### Study Population

This cohort study used data from the ECHO program from January 1, 1986, to December 31, 2015. Data were analyzed from January 1 to June 30, 2021. The ECHO program is a large collaborative consortium that seeks to investigate how environmental exposures in early life, including physical, chemical, social, behavioral, biological, natural, and built environments, affect child health and development. Briefly, ECHO comprises individual prebirth and birth cohorts across the US that have already enrolled participants. As detailed elsewhere,^20,21^ participating cohorts implemented the ECHO-wide Cohort Data Collection Protocol, which specifies the data elements not only for new or ongoing data collection but also for extant data to be uploaded onto an ECHO-wide cohort data platform. For the present study, we used extant data previously collected by individual cohorts that were already shared on the data platform. We included individual participants in the analytic sample if they had at least 1 anthropometric measure in the first 5 years of life and at least 1 measure of pubertal onset. Of 69 ECHO cohorts that participated in the program, we included 36 cohorts (n = 7495 participants) (eTable 1 in Supplement 1) who met the inclusion criteria and had available data in the data platform at the time of analyses. Parents or guardians provided written informed consent, and institutional review boards at each study site approved each local protocol. This study followed the Strengthening the Reporting of Observational Studies in Epidemiology (STROBE) reporting guideline.

### Exposure

In all participating cohorts, trained personnel obtained measures of weight and length (if younger than 2 years) or standing height (if 2 years or older) in children according to standardized protocols, from which we derived BMI. We examined child growth across 3 age periods in the first 5 years of life with potential etiological relevance to health outcomes in later years: early infancy (0-0.5 years), late infancy (0.5-2 years), and early childhood (2-5 years).^22^ We estimated annual individual-level rates of weight (kilograms), length or height (centimeters), and BMI gain across these age periods using linear spline mixed-effects models with knot points at ages 0.5 and 2 years. Details of the estimation procedure are shown in the eMethods in Supplement 1.

### Primary Outcome

Linear growth typically accelerates during puberty because of activation of the hypothalamic-pituitary axis, and the timing of the pubertal growth spurt (ie, age at peak height velocity [APHV]) can be used as an objective marker of pubertal timing.^23^ Hence, we considered APHV as the primary outcome. We estimated APHV in 34 cohorts (n = 5109) using longitudinal height data from both research visits and medical records (median [range] height measurements per child, 4 [2-19]; median [range] age, 8.4 [5-22] years). We fit participant-specific height growth curves using the Superimposition by Translation and Rotation growth model.^24^ Briefly, this model uses a shape-invariant natural cubic spline curve and a nonlinear random-effects model to estimate a population mean height growth curve for the entire sample and each participant’s deviation from the population mean curve as random effects. We identified the optimal model using the bayesian information criterion. We estimated APHV for each child by differentiating the individually predicted height curves and locating the maximum inflection point during adolescence where the derivative equals 0.

### Secondary Outcomes

Beginning at the earliest age of 6 years (range, 6.0-20.4 years), 10 cohorts (n = 3121) obtained self-reported pubic hair staging across 3 developmental periods (midchildhood: mean [SD] age, 8.3 [0.8] years; early adolescence: mean [SD] age, 11.7 [1.2] years; and late adolescence: mean [SD] age, 17.1 [1.2] years) in boys and girls (median [range] number of measures per child, 2 [1-4]) through questionnaires using the Tanner criteria for stage of maturation (stages 1-5) with descriptions accompanied by pictographs.^25,26^ We considered pubic hair stage greater than 1 as pubertal onset in accordance with previous studies.^17,27^

Beginning at the earliest age of 6 years (range, 6.1-21.2 years), 26 cohorts (n = 4969) evaluated pubertal onset across midchildhood, early adolescence, and late adolescence via a widely used and validated written Pubertal Development Scale (PDS)^28^ completed by parents (median [range] number of measures per child, 2 [1-7]). Previous studies have used the PDS at a similar early age, thus validating its use in our study.^29^ A recent study found that parent-reported PDS had strong internal consistency (Cronbach α = 0.94) and test-retest reliability (intraclass correlation coefficient, 0.88).^30^ Moreover, the PDS has been correlated with physician Tanner staging for breast development in girls^31^ and pubic hair staging in boys.^32^ Pubertal development scale questions for boys include 4 items: voice deepening, facial and body hair growth, acne, and growth spurt. Questions for girls include 5 items: breast development, body hair growth, acne, growth spurt, and menarche. The response options for each item (except menarche) were “not yet started” (1 point), “barely started” (2 points), “definitely started” (3 points), “seems complete” (4 points), and “I don’t know” (coded as missing). A “yes” answer on the menarche question received 4 points, whereas a “no” answer received 1 point. We derived a continuous puberty score ranging from 1 to 4 for each participant by summing the point values and averaging across all items. We then dichotomized the variable as prepubertal (puberty score equals 1) vs pubertal (puberty score greater than 1) in accordance with a previous study.^33^

Beginning at the earliest age of 6 years (range, 6.1-21.2 years), 26 cohorts (n = 1559) obtained repeated data on age at menarche for girls from a series of either parent- or child-reported questionnaires. Briefly, mothers indicated whether their daughters—or girls self-reported—whether they had attained their first menstrual period and the month and year of its occurrence.

### Covariates

We obtained information on the following maternal characteristics from maternal or caregiver reports or medical records: maternal age at delivery (in years); educational level during pregnancy (less than 4-year college degree or 4-year college degree or higher); annual household income during pregnancy (<$75 000 or ≥$75 000); prenatal cigarette smoking (yes or no); whether the mother was nulliparous; whether the mother had gestational diabetes, hypertension, or preeclampsia; mode of delivery (vaginal or cesarean); prepregnancy BMI; gestational weight gain (kilograms); and gestational age at delivery (in weeks). In addition, the following pediatric characteristics were obtained from caregiver reports or medical records: child’s birth year (before 2000, 2000-2010, or after 2010), sex (boy or girl), self-reported race (American Indian or Alaska Native, Asian, Black, Native Hawaiian or Pacific Islander, White, multiple races, or other race [the other race category was obtained from a survey questionnaire with no further breakdown available]), and Hispanic ethnicity (yes or no). Because of the small sample size, we combined the number of children who identified as American Indian or Alaska Native, Native Hawaiian or Pacific Islander, multiple races, or other race into a single category. We selected these covariates based on previous publications that found an association between child growth and pubertal onset.^34,35,36,37,38,39,40^

### Statistical Analyses

We decided a priori to conduct all analyses separately in boys and girls given the known sex differences in pubertal onset.^41^ We used multivariable regression models to estimate mean differences in APHV by growth periods. We used repeated measures of Tanner staging for pubic hair development, puberty score, and menarche to ascertain the time to events of a pubic hair stage greater than 1, puberty score greater than 1, and menarche. However, we were unable to ascertain the exact age at which a child attained a Tanner stage greater than 1 for pubic hair development or a puberty score greater than 1. We were able to ascertain only what pubic hair stage or puberty score was reported at the time each questionnaire was completed. Thus, the event (ie, pubic hair stage greater than 1 or puberty score greater than 1) may have been attained sometime during the interval between 2 questionnaires. To address this issue, we treated the time to pubic hair stage greater than 1 and time to puberty score greater than 1 as arbitrarily censored event times and considered the data left censored if the event was attained by the first questionnaire, interval censored if the event was attained sometime between 2 questionnaires, and right censored if the event was not attained by the last visit, in accordance with previous studies.^42,43^ We then used parametric survival models with exponential distribution to estimate associations of growth velocities in early infancy, late infancy, and early childhood with time to pubic hair stage greater than 1, puberty score greater than 1, and menarche, respectively. Examining these pubertal onset markers as time-to-event outcomes makes better use of the longitudinal measures, increases statistical power, and avoids bias arising from excluding children who have not yet achieved the event at each visit or by the last follow-up visit.

We analyzed growth at each age period in separate regression models. For all analyses, we adjusted for the child’s birth year, race, and Hispanic ethnicity and maternal age at delivery; educational level; annual household income during pregnancy; prenatal cigarette smoking; whether the mother was nulliparous; whether the mother had gestational diabetes, hypertension, or preeclampsia; mode of delivery; prepregnancy BMI; gestational weight gain; and gestational age at delivery. We also adjusted for birth size and growth in all previous age periods but not in subsequent age periods; for example, when examining the association between weight velocity in late infancy and pubertal onset markers, we adjusted for birth weight and weight velocity in early infancy but not weight velocity in early childhood. This approach reduces the likelihood of collider-stratification bias, which may occur when controlling for subsequent growth periods that lie on the causal pathway^44^ (eFigure in Supplement 1). We also accounted for clustering of children from the same cohort by including a random-effect term for cohort. We examined effect modification by child race or Hispanic ethnicity, year of birth, and maternal educational level by stratifying all analyses according to these variables. eTable 2 in Supplement 1 summarizes the analytic models used in this study.

We used multiple imputation by chained equations^45^ to impute values for missing covariates. Briefly, multiple imputation generates multiple predictions for each missing value derived from distributions of and associations among observed variables in the data set. Using multiple plausible values would not only quantify the uncertainty in the imputations but also yield more accurate SEs, reducing the likelihood of spurious results.^46^ We chose this method over single-value imputation methods, which often fail to account for uncertainty in imputing missing values, do not use all available information, can introduce bias, and artificially increase precision.^47^ We generated 100 imputed data sets for all 7495 children with available growth measures in the first 5 years of life and at least 1 pubertal outcome. The imputation model included the primary outcome (ie, APHV) as well as all exposures and covariates under study. We performed all analyses using R software, version 3.6.2 (R Foundation for Statistical Computing) and defined statistical significance as α = .05.

## Results

Of 7495 children included in the analysis, 3772 (50.3%) were girls and 3723 (49.7%) boys; 868 (11.6%) self-reported as American Indian or Alaska Native, Native Hawaiian or Pacific Islander, multiple races, or other race, 120 (1.6%) as Asian, 1705 (22.7%) as Black, 1124 (15.0%) as Hispanic, and 4505 (60.1%) as White individuals; and 6307 (84.1%) were born during or after the year 2000. The mean (SD) maternal age at delivery was 29.2 (6.1) years, and mean (SD) prepregnancy BMI of mothers was 26.5 (6.7) (Table 1). Velocities of weight and length or height gain in children monotonically declined through the first 5 years of life, with boys having faster gains in weight, length or height, and BMI in early infancy compared with girls (eTable 3 in Supplement 1). As expected, girls had a younger APHV (10.8 vs 12.9 years in boys), higher pubertal score in childhood and adolescence, and higher prevalence of Tanner pubic hair stage greater than 1 compared with boys (eTable 4 in Supplement 1). Mean (SD) age at menarche was 13.2 (2.9) years among girls who reported achieving menarche. As expected, in both boys and girls, APHV was negatively correlated with puberty score (r = −0.01 to −0.28) and Tanner staging for pubic hair development (r = −0.01 to −0.18), whereas puberty score was positively correlated with Tanner staging for pubic hair (r = 0.04 to 0.70). In girls, age at menarche was positively correlated with APHV (r = 0.03) and negatively correlated with puberty score and Tanner staging for pubic hair (r = −0.05 to −0.34) (eTable 5 in Supplement 1).

**Table 1.** Characteristics of Study Participants in Analytic Sample

Characteristics	No. (%) (n = 7495)
Maternal characteristics	
Age at delivery (n = 7320), mean (SD), y	29.2 (6.1)
Educational level during pregnancy	
<4-y College degree	2151 (28.7)
≥4-y College degree	2223 (29.7)
Missing	3121 (41.6)
Annual household income during pregnancy, $	
<75 000	2393 (31.9)
≥75 000	512 (6.8)
Missing	4590 (61.2)
Prenatal cigarette smoking	
Yes	683 (9.1)
No	4409 (58.8)
Missing	2403 (32.1)
Nulliparous	
Yes	1306 (17.4)
No	2033 (27.1)
Missing	4156 (55.5)
Gestational diabetes	
Yes	215 (2.9)
No	3596 (48.0)
Missing	3684 (49.2)
Gestational hypertension/preeclampsia	
Yes	401 (5.4)
No	4196 (56.0)
Missing	2898 (38.7)
Mode of delivery	
Vaginal	3620 (48.3)
Cesarean	1495 (19.9)
Missing	2380 (31.8)
Prepregnancy BMI (n = 5032), mean (SD)	26.5 (6.7)
Gestational weight gain (n = 4116), mean (SD), kg	15.5 (10.5)
Gestational age at delivery (n = 6527), mean (SD), wk	38.2 (3.3)
Child characteristics	
Birth year	
Before 2000	1188 (15.9)
2000-2010	3884 (51.8)
After 2010	2423 (32.3)
Sex	
Male	3723 (49.7)
Female	3772 (50.3)
Race	
American Indian or Alaska Native, Native Hawaiian or Pacific Islander, multiple races, or other race^a^	868 (11.6)
Asian	120 (1.6)
Black	1705 (22.7)
White	4505 (60.1)
Missing	297 (4.0)
Hispanic ethnicity	1124 (15.0)
Missing	29 (0.4)

Abbreviation: BMI, body mass index (calculated as weight in kilograms divided by height in meters squared).

^a^ The other race category was obtained from a survey questionnaire with no further breakdown available.

### Association of Early-Life Growth With Pubertal Onset in Boys

After adjusting for maternal and child confounders, faster weight gain (per 1-SD increase) in early infancy (β, −0.08 years; 95% CI, −0.10 to −0.06), late infancy (β, −0.10 years; 95% CI, −0.12 to −0.08), and early childhood (β, −0.07 years; 95% CI, −0.08 to −0.05) was associated with younger APHV. Similar associations were noted for velocities of length or height (β −0.07 to −0.11 years) and BMI gains (β, −0.03 to −0.04 years) during the same age periods. There was no evidence of effect modification by child race or Hispanic ethnicity, maternal educational level, or year of birth. No associations were observed between faster weight, length or height, or BMI gains in all age periods and time to puberty score greater than 1 or time to pubic hair stage greater than 1 (Table 2).

**Table 2.** Associations of Velocities of Weight, Length or Height, and BMI Gain in Early Infancy, Late Infancy, and Early Childhood With Pubertal Onset in Boys

	Age at peak height velocity, y, β (95% CI)^a^^,^^b^	Hazard ratio (95% CI)^b^	
		Risk of earlier time to puberty score >1^a^	Risk of earlier time to pubic hair stage >1^a^
**Velocity of weight gain**			
Early infancy	−0.08 (−0.10 to −0.06)	1.01 (0.96 to 1.08)	1.00 (0.96 to 1.04)
Late infancy	−0.10 (−0.12 to −0.08)	1.00 (0.98 to 1.02)	1.00 (0.95 to 1.04)
Early childhood	−0.07 (−0.08 to −0.05)	1.00 (0.93 to 1.06)	1.02 (0.98 to 1.06)
**Velocity of length or height gain**			
Early infancy	−0.11 (−0.13 to −0.09)	0.99 (0.93 to 1.04)	1.03 (0.98 to 1.08)
Late infancy	−0.10 (−0.12 to −0.09)	1.00 (0.98 to 1.01)	1.01 (0.98 to 1.05)
Early childhood	−0.07 (−0.08 to −0.05)	1.00 (0.98 to 1.03)	0.99 (0.96 to 1.03)
**Velocity of BMI gain**			
Early infancy	−0.03 (−0.05 to −0.01)	1.01 (0.99 to 1.03)	0.96 (0.93 to 1.00)
Late infancy	−0.04 (−0.06 to −0.02)	1.01 (0.97 to 1.05)	0.99 (0.98 to 1.01)
Early childhood	−0.03 (−0.04 to −0.01)	0.98 (0.96 to 1.00)	1.02 (0.99 to 1.05)

Abbreviation: BMI, body mass index (calculated as weight in kilograms divided by height in meters squared).

^a^ Adjusted for child’s birth year, race, and Hispanic ethnicity and maternal age at delivery; educational level during pregnancy; annual household income during pregnancy; prenatal cigarette smoking; whether mother was nulliparous; whether mother had gestational diabetes, hypertension, or preeclampsia; mode of delivery; prepregnancy BMI; gestational weight gain; and gestational age at delivery.

^b^ All effect estimates reflect a 1-SD increase in velocities of weight, length or height, or BMI gain.

### Association of Early-Life Growth With Pubertal Onset in Girls

After adjusting for maternal and child confounders, faster weight gain per 1-SD increase (β, −0.03 years; 95% CI, −0.05 to −0.01) and length or height gain per 1-SD increase (β, −0.02 years; 95% CI, −0.04 to 0.00) in early childhood only was associated with younger APHV. No associations with APHV were observed for velocities of BMI gain at any age period. Similarly, growth velocities in all age periods were not related to time to puberty score greater than 1. Faster weight gain per 1-SD increase (hazard ratio [HR], 1.04; 95% CI, 1.01-1.07) and BMI gain per 1-SD increase (HR, 1.04; 95% CI, 1.01-1.07) in early childhood were associated with earlier time to pubic hair stage greater than 1, whereas a faster BMI gain per 1-SD increase in late infancy was associated with earlier time to menarche (HR, 1.14; 95% CI, 1.05-1.24) (Table 3). These associations were not modified by maternal educational level, child race or Hispanic ethnicity, or year of birth. eTable 6 in Supplement 1 provides a qualitative summary of our study findings.

**Table 3.** Associations of Velocities of Weight, Length or Height, and BMI Gain in Early Infancy, Late Infancy, and Early Childhood With Pubertal Onset in Girls

	Age at peak height velocity, y, β (95% CI)^a^^,^^b^	Hazard ratio (95% CI)^b^		
		Risk of earlier time to puberty score >1^a^	Risk of earlier time to pubic hair stage >1^a^	Risk of earlier time to menarche^a^
**Velocity of weight gain**				
Early infancy	0.02 (−0.01 to 0.04)	0.99 (0.93 to 1.05)	1.03 (0.99 to 1.08)	0.92 (0.83 to 1.01)
Late infancy	0.02 (−0.003 to 0.04)	1.01 (0.98 to 1.03)	1.00 (0.98 to 1.02)	1.07 (0.99 to 1.05)
Early childhood	−0.03 (−0.05 to −0.01)	1.01 (0.96 to 1.06)	1.04 (1.01 to 1.07)	1.07 (0.94 to 1.21)
**Velocity of length or height gain**				
Early infancy	0.01 (−0.02 to 0.03)	0.99 (0.94 to 1.04)	1.05 (1.00 to 1.10)	0.90 (0.84 to 1.00)
Late infancy	0.04 (0.02 to 0.06)	0.99 (0.97 to 1.02)	1.00 (0.96 to 1.05)	0.89 (0.82 to 1.00)
Early childhood	−0.02 (−0.04 to 0.00)	1.01 (0.99 to 1.04)	1.02 (0.98 to 1.07)	1.02 (0.93 to 1.12)
**Velocity of BMI gain**				
Early infancy	0.01 (−0.02 to 0.03)	1.02 (0.99 to 1.04)	0.99 (0.96 to 1.01)	1.01 (0.94 to 1.08)
Late infancy	0.001 (−0.02 to 0.03)	1.02 (0.99 to 1.04)	1.00 (0.95 to 1.06)	1.14 (1.05 to 1.24)
Early childhood	−0.002 (−0.02 to 0.02)	0.98 (0.96 to 1.00)	1.04 (1.01 to 1.07)	0.99 (0.89 to 1.11)

Abbreviation: BMI, body mass index (calculated as weight in kilograms divided by height in meters squared).

^a^ Adjusted for child’s birth year, race, and Hispanic ethnicity and maternal age at delivery; educational level during pregnancy; annual household income during pregnancy; prenatal cigarette smoking; whether mother was nulliparous; whether mother had gestational diabetes, hypertension, or preeclampsia; mode of delivery; prepregnancy BMI; gestational weight gain; and gestational age at delivery.

^b^ All effect estimates reflect a 1-SD increase in velocities of weight, length or height, or BMI gain.

## Discussion

In this nationwide multicohort study, we observed sex-specific associations of early-life growth with pubertal onset. Boys with faster gains in weight, length or height, and BMI in early infancy, late infancy, and early childhood exhibited earlier pubertal onset evidenced by a younger APHV. The strength of these associations was similar across all age periods. In girls, there appeared to be age periods during which early-life growth was associated with pubertal onset. Those with faster gains in weight and length or height in early childhood only had younger APHV, whereas those with faster gains in weight and BMI in late infancy and early childhood exhibited earlier time to menarche and pubic hair stage greater than 1, respectively. Although we have no clear biological explanation for this observation, previous reports have noted associations between higher prepubertal BMI and more advanced pubic hair development in girls than in boys.^48,49^

Our findings are in line with previous studies that have examined associations between child growth and pubertal onset. A study of Afro-Caribbean children reported that rapid weight gain from birth to 6 months of age was associated with more advanced pubic hair development in adolescent girls and higher testicular volume in adolescent boys.^34^ Similar results have been reported in other studies with longer age intervals, including from birth to 12 months in the Danish National Birth Cohort^35^ and the Birth to Twenty cohort,^39^ from birth to 20 months in the Avon Longitudinal Study of Parents and Children,^38^ and from birth to 24 months in the Dortmund Nutritional and Anthropometric Longitudinally Designed study.^36^ Findings from the North Carolina Infant Feeding study also showed that greater weight gains during infancy and early childhood were associated with earlier menarche and more advanced pubic hair development.^40^ The clinical implications of earlier menarche are well known; it has been shown to increase the risks of breast cancer and coronary heart disease in adulthood.^50,51^ However, less is known about the implications of younger APHV and time to pubic hair stage greater than 1, although the use of growth trajectory hallmarks as markers of future disease risk have been discussed.^52^ Previous studies have reported that a 1-year difference in APHV is associated with hormonal changes that are associated with cancer risk^53^ and cardiovascular disease mortality in adulthood.^54^ Although the present study found relatively small differences in APHV among children with faster growth rates in infancy and early childhood, these differences may still have similar, albeit smaller, health consequences.

Taken together, findings of the present study suggest that faster growth during the first 5 years of life could be a potential indicator to identify children who are likely to experience earlier pubertal onset. Many interrelated mechanisms may be at play. Previous studies have shown that rapid weight gain in early life has been associated with elevated insulinlike growth factor 1 concentrations,^55^ insulin resistance,^56,57^ adipokines (eg, leptin),^58^ and adrenal androgen concentrations,^59^ all of which could promote the activity of the gonadotropin-releasing hormone pulse generator,^9^ consequently altering the timing of puberty. We speculate that insulinlike growth factor 1 may be a factor in the associations observed in the present study, either directly or indirectly through sex steroid synthesis and secretion. Alternatively, in girls, androgens and adipokines may be factors in the observed associations for pubic hair staging and menarche, respectively. Such biological mechanisms, however, have yet to be fully elucidated. Genetics might also contribute to the observed associations, as some children may have an underlying predisposition to both grow rapidly and attain sexual maturity quickly.^60^

Despite these observations, it should be noted that such growth patterns are not exposures in the true sense. Growth during these age periods is likely the result of exposure to other factors related to growth and body composition (eg, upstream social factors, diet and other environmental exposures, and physical activity), and these exposures most likely affect health status later in life.^61,62^

The novel contributions of our study to the extant literature are also worth noting. Previous US reports examining pubertal characteristics have mostly been based on children born in the 1960s through the 1980s,^63^ a time period before the obesity epidemic began and when environmental factors were comparatively favorable for growth.^64^ Moreover, a recent review highlighted that most studies assessed anthropometric measures too late in childhood to capture growth before onset of puberty,^65^ thus making it difficult to elucidate the temporal order of the association. Most of the existing research on pubertal onset has also focused on age at menarche only,^13^ leaving important gaps in the understanding of the contributing factors of pubertal onset in boys and the development and progression of secondary sexual characteristics (eg, pubic hair development) in boys and girls. The present study directly addresses these key research and knowledge gaps by (1) examining a contemporary cohort of children born and raised mostly in the first decade of the 21st century, amid the evolution of the obesity epidemic^66^; (2) focusing on growth during a period when puberty is still far in the horizon, which reduces the likelihood of reverse causation and allows us to better elucidate the temporal order of the association between growth and puberty; (3) analyzing repeated measures of secondary sexual characteristics from midchildhood through adolescence; and (4) examining the APHV as a measure of pubertal timing in boys and girls, which has been shown to be an objective marker of pubertal onset.^67^

### Strengths and Limitations

This study has strengths. They include the large sample size, prospective study design, long-term follow-up, and wide range of covariates obtained by highly trained staff using standardized protocols. Furthermore, children in our sample were distributed across diverse geographic regions in the US, which not only makes this study nationally representative but also improves generalizability.

This study also has limitations. First, we used child-reported measures of pubic hair staging and parent-reported measures of pubertal score, which may be more prone to error and misclassification than information collected by trained observers or physicians during clinical examinations. Although clinical assessment of pubertal onset is considered the criterion standard, it is still dependent on observer training and experience.^68^ Furthermore, a recent review of pubertal assessment methods highlighted the low acceptability of clinical examination in a research setting.^69^ By using parent- and child-reported information on pubertal onset, we were able to conduct a larger population-based study examining the association between early-life growth and pubertal onset, albeit at the cost of some measurement error. Moreover, in large, population-based studies, self-assessment of secondary sexual characteristics (including pubic hair development) performs similarly well to assessments by trained physicians when both are compared against objective biomarkers of pubertal onset,^68^ suggesting that the cost in terms of measurement error may be minor.

Second, because we do not have a measure of maternal age at menarche—a strong factor in offspring pubertal onset^70^ as well as growth in early life^71^—we cannot rule out the possibility of residual confounding in the observed associations. We did, however, adjust the analyses for prepregnancy BMI—a potential mediating variable of the association between maternal age at menarche and offspring early-life growth and puberty—which might have minimized confounding by maternal age at menarche.

Third, the use of BMI is currently not recommended for children younger than age 2 years; rather, weight-for-length (WFL) is recommended as an indicator of growth for children in this age group.^72^ Previous studies, however, identified high concordance between WFL and BMI in infancy.^73^ Moreover, the choice of WFL or BMI as indicators of growth in children younger than 2 years has not been found to substantially affect the ability to estimate cardiometabolic outcomes in adolescence.^74^ These observations suggest that the use of BMI is likely to yield comparable results to those of WFL for children younger than age 2 years.

## Conclusions

The findings of this cohort study suggest that there are sex-specific associations of faster growth in early life with earlier pubertal onset. The most clinically relevant implication of these findings is that children with faster gains in weight, length or height, or BMI immediately after birth, during late infancy, or in early childhood may need to be monitored closely for earlier onset of puberty and referred as appropriate for supportive services. In the long term, results of the present study may inform future research that aims to develop and/or test preventive interventions to optimize nutrition, environmental exposures, physical activity, and other behaviors related to growth during these age periods.

## Notes

Supplement 1. **eTable 1.** List of Cohorts Included in Analytic Sample **eTable 2.** Description of the Different Analytic Models Used in This Study **eTable 3.** Estimated Velocities of Weight, Length or Height, and BMI in the First 5 Years of Life **eTable 4.** Characteristics of Pubertal Development Markers **eTable 5.** Spearman Correlations of Pubertal Development Markers **eTable 6.** Qualitative Summary of Study Findings **eFigure.** Directed Acyclic Graph Representing the Possible Relationship Among Baseline Covariates, Child Growth, and Pubertal Outcomes **eMethods.** Procedure for Estimating Growth Velocities in the First 5 Years of Life
eReferences

Supplement 2.
Nonauthor Collaborators
