# Supplementary material for: Analysis of Early-Life Growth and Age at Pubertal Onset in US Children
Source: JAMA Netw Open. 2022 Feb 4;5(2):e2146873. doi: 10.1001/jamanetworkopen.2021.46873 (PMC8817204; doi:10.1001/jamanetworkopen.2021.46873)
Supplement: Supplement 1. — eTable 1. List of Cohorts Included in Analytic Sample eTable 2. Description of the Different Analytic Models Used in This Study eTable 3. Estimated Velocities of Weight, Length or Height, and BMI in the First 5 Years of Life eTable 4. Characteristics of Pubertal Development Markers eTable 5. Spearman Correlations of Pubertal Development Markers eTable 6. Qualitative Summary of Study Findings eFigure. Directed Acyclic Graph Representing the Possible Relationship Among Baseline Covariates, Child Growth, and Pubertal Outcomes eMethods. Procedure for Estimating Growth Velocities in the First 5 Years of Life eReferences [file jamanetwopen-e2146873-s001.pdf]

Supplementary Online Content

Aris IM, Perng W, Dabelea D, et al; Program Collaborators for Environmental Influences on Child Health Outcomes. Analysis of early-life growth and age at pubertal onset in US children. *JAMA Netw Open*. 2022;5(2):e2146873.
doi:10.1001/jamanetworkopen.2021.46873

eTable 1. List of Cohorts Included in Analytic Sample
eTable 2. Description of the Different Analytic Models Used in This Study
eTable 3. Estimated Velocities of Weight, Length or Height, and BMI in the First 5 Years of Life
eTable 4. Characteristics of Pubertal Development Markers
eTable 5. Spearman Correlations of Pubertal Development Markers
eTable 6. Qualitative Summary of Study Findings
eFigure. Directed Acyclic Graph Representing the Possible Relationship Among Baseline Covariates, Child Growth, and Pubertal Outcomes
eMethods. Procedure for Estimating Growth Velocities in the First 5 Years of Life
eReferences.

This supplementary material has been provided by the authors to give readers additional information about their work.

© 2022 Aris IM et al. *JAMA Network Open.*

eTable 1. List of Cohorts Included in Analytic Sample

	**Cohort Name**	**Description**	**N included in analysis**
1	NICU Hospital Exposures and Long-Term Health	A longitudinal study that capitalizes on the infrastructure, biorepositories, and extensive clinical databases of four existing preterm infant study cohorts to examine the sources and impact of NICU-based phthalate and other potentially harmful exposures on their neurodevelopment, lung health, growth and pubertal development in childhood.	21
2	Developmental Impact of NICU exposures - PROP, TOLSURF, PENUT	A longitudinal study that capitalizes on the infrastructure, biorepositories, and extensive clinical databases of four existing preterm infant study cohorts to examine the sources and impact of NICU-based phthalate and other potentially harmful exposures on their neurodevelopment, lung health, growth and pubertal development in childhood.	219
3	Healthy Start	A large cohort of ethnically diverse mother-infant dyads who were followed until the child was 3 years of age, and continues follow-up on the mothers and children as the child reaches 4-6 years of age.	280
4	BAMBAM	Longitudinal study of normal brain and behavioral development in more than 500 children recruited between birth and 5 years of age.	187
5	Boricua Youth Study	Longitudinal intergenerational cohort studying Puerto Rican community; enrolled 2,500 children aged 5-13 (Generation 1) in two sites (South Bronx NY and San Juan PR) and ongoing enrollment in Generation 2. SRS administered in Generation 2 at ages 3-10.	65
6	Safe Passage Study	Longitudinal study of impact of >4400 children examining the impact prenatal and early childhood environmental exposures on later health outcomes among rural and American Indian youth.	151
7	Pregnancy Environment and Lifestyle Study	A longitudinal birth cohort of 3,350 mother-infant pairs, investigating the associations between phenol exposures in first and second trimesters and GDM risk and the related outcome of infant macrosomia.	2
8	Kaiser Permante Research Bank	A genetic epidemiology population resource which integrates data from multiple sources from consenting Kaiser Permanante North Carolina adult members, including clinical data from electronic health records, lifestyle and risk factor data from surveys, and environmental exposure data from both laboratory and geographic information systems.	1
9	Childhood Origins of Asthma Study	A "high-risk" (at least one parent with asthma or allergies) birth cohort study that aims to examine the relationships between environmental factors including respiratory illnesses, genetic factors, immune system factors, and the development of asthma.	243
10	Urban Environment and Childhood Asthma	A longitudinal study of children from urban areas in economically disadvantaged neighborhoods in New York City, Boston, Baltimore, and St. Louis, with the aim of identifying insight into the unique effects of the inner city environment on the development of asthma	468
11	Cincinnati Childhood Allergy & Air Pollution Study	A prospective cohort study of 762 newborns living either within 400 m of interstate highways, exposed, or further than 1 km, unexposed and followed up to age 4 and age 7. The goal of the newborn cohort study was to prospectively evaluate whether exposure to diesel exhaust increases sensitization to aeroallergens.	76
12	Infant Susceptibility to Pulmonary Infections and	A population-based cohort of 1952 infants who will be followed to age 6-8 years, when asthma will be defined	15

© 2022 Aris IM et al. *JAMA Network Open.*

	Asthma Following RSV Exposure		
13	Epidemiology of Home Allergens and Asthma Study	A high-risk (parental asthma and/or allergy) birth cohort from Greater Boston that aims to evaluate influences of early-life indoor home exposures to allergens, fungi, and microbial components on the development of wheeze, asthma, lung function, and related immune responses	370
14	Childhood Allergy/Asthma Study	Birth cohort enrolled from 4/15/1987 until 8/31/1989. Annual survey followed by clinic visit at 6-7 years of age and last follow up at 18 years of age.	350
15	ReCHARGE	Longitudinal follow-up extension of the California CHARGE case-control study, including autism sepctrum disorder (ASD) cases, developmental delay (DD) controls, and typically developing controls. ASD and DD groups identified through the CA Department of Developmental Disabilities and confirmed according to clinical assessment.	432
16	New Hampshire Birth Cohort Study	Prospective cohort enrolling pregnancies from NH clinics since 2009, with private household water systems. SRS-2 administered in early and middle childhood.	411
17	CANDLE study	A prospective study of 1385 mother-child dyads at four hospitals in Memphis, TN, with the goal of understanding the early-life predictors of child socioemotional and neurocognitive development	809
18	Global Alliance to Prevent Prematurity and Stillbirth	A data and tissue bio-bank to be used to research normal and abnormal pregnancies, including how pregnancy affects maternal and child health after deliver.	11
19	ECHO-NOVI	A cohort of 1060 infants born <30 wks postmenstrual age during their NICU stay at 7 sites participating in the Vermont-Oxford Network (VON). The goal of this study is to determine which infants born <30 wks PMA are at greatest risk for impaired development using a neurobehavioral assessment and a medical risk score	57
20	Early Growth and Development Study Pediatric Cohort	A longitudinal prospective adoption study of biological parents, adoptive parents, and adopted children, with the aim to examine how family, peer and contextual processes affect children's adjustment	901
21	Vitamin D Antenatal Asthma Reduction Trial	A randomized, double-blind, placebo-controlled study of vitamin D (daily 4,000 IU vitamin D plus a multivitamin with 400 IU vitamin D) vs. placebo (daily placebo pill plus a multivitamin with 400 IU vitamin D) in pregnant women	432
22	Vitamin C to Decrease Effects of Smoking in Pregnancy on Infant Lung Function	A randomized trial to assess whether Vitamin C supplementation (500 mg/day) during pregnancy will block the adverse effects of maternal smoking on infant pulmonary function measured at 3 months of age (forced expiratory flows) in infants born to smoking mothers.	61
23	In-Utero Smoke, Vitamin C, and Newborn Lung Function	A randomized controlled trial of pregnant smokers to vitamin C supplementation versus placebo during pregnancy	108
24	University of California Davis - BRSC	Multi-site study enrolling younger siblings (<9, 12, or 18 months of age depending on site) of a child with ASD.	19
25	Markers of Autism Risk in Babies - Learning Early Signs	A longitudinal study for pregnant women who have a biological child with autism spectrum disorder, with the aim of investigating possible pre-natal and post-partum biological and environmental exposures and risk factors that may contribute to the development of autism.	14

© 2022 Aris IM et al. *JAMA Network Open.*

26	Infant Brain Imaging Study	Multi-site study enrolling younger siblings of a child with ASD. Low-risk comparison families were also enrolled	30
27	Early Autism Risk Longitudinal Investigation	High-familial risk study that enrolled pregnant mothers at <28 weeks gestation who had a previous child with ASD from 4 US sites (Northern CA and the San Francisco Bay Area; MD; and PA), and following the younger sibling's development from birth.	29
28	Project Viva	Women recruited from Boston, MA-area obstetric practices in first trimester of singleton pregnancy 1999-2002 and followed thereafter.	657
29	Extremely Low Gestational Age Newborn	Cohort including 1,506 extremely preterm neonates (<28 weeks gestation) and their mothers enrolled from 2002-2004 at 14 institutions in 5 US states (CT, IL, MA, MI, NC).	34
30	Columbia Center for Children's Environmental Health (CCCEH) Sibling Cohort	A cohort of 129 younger siblings of the children from the CCCEH Mothers and Newborns cohort, examining the impact of prenatal environmental exposures on neurodevelopmental and other health outcomes	32
31	Utah Childrens Project	A longitudinal cohort of couples seeking pregnancy to assess exposures in the periconceptional, embryonic and prenatal periods, and their relationship to subsequent child health, growth and development	313
32	Asthma Coalition on Community, Environment & Stress	A prospective cohort of prenatally enrolled mother-child pairs designed to study the interactive effects of early life stress and other physical environmental factors on urban childhood asthma risk	5
33	PRogramming of Intergenerational Stress Mechanisms	A longitudinal pregnancy cohort to test the impact of maternal and child pre- and postnatal stress exposures on maternal prenatal and child stress regulation and on child health outcomes in infancy and early childhood.	82
34	Inova Childhood Longitudinal Study/ George Mason ECHO Cohort	A longitudinal pregnancy/ birth cohort nested within a healthcare system in Northern Virginia, with the aim of conducting genomic analyses on prenatal and delivery biobanking samples to facilitate gene-environment interaction research.	31
35	The Infant Development and Environment Study	Prospective pregnancy cohort examining environmental exposures and infant/child development; enrolled approximately 800 mother-child pairs across four institutes (UC San Francisco, University of Rochester Medical Center, University of Michigan, and University of Washington/Seattle Children's Hospital) between 2010-2012. SRS administered in childhood.	239
36	CCCEH Mothers and Newborns study	A prospective low-income cohort of African American and Dominican women from northern Manhattan and the South Bronx designed to examine the role of environmental exposures on birth outcomes	340

© 2022 Aris IM et al. *JAMA Network Open.*

eTable 2. Description of the Different Analytic Models Used in This Study

Exposure	***Analytic model***
. Early infancy growth	Linear spline mixed-effects models with maximum likelihood estimation
. Late infancy growth	
. Early childhood growth	
Outcomes	***Analytic model***
. Age at peak height velocity	SITAR growth model
. Pubic hair staging	Parent- or child-reported questionnaires
. Pubertal score	
. Age at menarche	
Statistical analyses	***Analytic model***
. Age at peak height velocity	Linear regression, adjusted for covariates
. Time to pubic hair stage>1	Parametric survival models with exponential distribution, adjusted for covariates
. Time to puberty score>1	
. Time to menarche	

SITAR: Superimposition by Translation and Rotation

© 2022 Aris IM et al. *JAMA Network Open.*

eTable 3. Estimated Velocities of Weight, Length or Height, and BMI in the First 5 Years of Life

	**Males**	**Females**
**Estimated velocity of weight gain (kg/year)**	**n=3723**	**n=3772**
. Early infancy	9.5 (1.7)	8.0 (1.8)
. Late infancy	3.2 (0.7)	3.3 (0.6)
. Early childhood	2.4 (0.6)	2.5 (0.6)
**Estimated velocity of length/height gain (cm/year)**	**n=3417**	**n=3470**
. Early infancy	34.5 (3.5)	31.7 (3.5)
. Late infancy	12.7 (1.4)	13.2 (1.4)
. Early childhood	8.0 (0.6)	8.2 (0.6)
**Estimated velocity of BMI gain (kg/m^2/year)**	**n=3392**	**n=3449**
. Early infancy	9.5 (2.5)	8.6 (2.4)
. Late infancy	-0.7 (0.5)	-0.5 (0.5)
. Early childhood	-0.2 (0.3)	-0.2 (0.3)

© 2022 Aris IM et al. *JAMA Network Open.*

eTable 4. Characteristics of Pubertal Development Markers

	Males		Females	
	**n**	**Mean (SD) or %**	**n**	**Mean (SD) or %**
Age at peak height velocity (years)	2625	12.9 (0.4)	2484	10.8 (0.5)
Puberty score 6–<10 years (units)	1164	1.46 (0.60)	1183	1.47 (0.59)
Puberty score 10–15 years (units)	913	1.86 (0.60)	945	2.21 (1.07)
Puberty score >15 years (units)	931	3.21 (0.49)	1277	3.80 (0.31)
Tanner pubic hair stage 6–<10 years	697		655	
. 1		75.0		66.3
. 2		18.4		26.1
. 3		5.2		5.7
. 4		0.9		1.8
. 5		0.6		0.2
Tanner pubic hair stage 10–15 years	733		740	
. 1		39.3		31.4
. 2		34.1		29.2
. 3		14.3		17.4
. 4		9.4		15.5
. 5		2.9		6.5
Tanner pubic hair stage >15 years	606		192	
. 1		0.0		0.5
. 2		0.3		0.5
. 3		3.6		2.6
. 4		31.7		30.2
. 5		64.4		66.1
Age at menarche (years)	–	–	1220	13.2 (2.9)

© 2022 Aris IM et al. *JAMA Network Open.*

eTable 5. Spearman Correlations of Pubertal Development Markers

**Males**	**APHV**	**Puberty score 6–<10y**	**Puberty score 10−15y**	**Puberty score >15y**	**Tanner pubic hair stage 6–<10y**	**Tanner pubic hair stage 10−15y**	**Tanner pubic hair stage >15y**	
**APHV**	1							
**Puberty score 6–<10y**	-0.01	1						
**Puberty score 10−15y**	-0.28*	0.27*	1					
**Puberty score >15y**	-0.18*	-0.29*	0.15*	1				
**Tanner pubic hair stage 6–<10y**	-0.01	0.44*	–	–	1			
**Tanner pubic hair stage 10−15y**	-0.15*	0.24*	0.51*	0.08	0.39*	1		
**Tanner pubic hair stage >15y**	-0.12*	-0.16*	0.04	0.46*	–	-0.07	1	
**Females**	**APHV**	**Puberty score 6–<10y**	**Puberty score 10−15y**	**Puberty score >15y**	**Tanner pubic hair stage 6–<10y**	**Tanner pubic hair stage 10−15y**	**Tanner pubic hair stage >15y**	**Age at menarche**
**APHV**	1							
**Puberty score 6–<10y**	-0.22*	1						
**Puberty score 10−15y**	-0.15*	0.45*	1					
**Puberty score >15y**	-0.12*	-0.06	-0.57*	1				
**Tanner pubic hair stage 6–<10y**	-0.11*	0.38*	–	–	1			
**Tanner pubic hair stage 10−15y**	-0.18*	0.46*	0.70*	-0.01	0.65*	1		
**Tanner pubic hair stage >15y**	0.01	-0.16*	0.06	0.25*	–	0.46*	1	
**Age at menarche**	0.03	-0.17*	-0.34*	-0.05	0.87*	-0.19*	-0.22*	1

*p<0.05.

© 2022 Aris IM, et al. *JAMA Netw Open.*

eTable 6. Qualitative Summary of Study Findings

	**Age at peak height velocity (APHV)**		**Risk of earlier time to puberty score>1**		**Risk of earlier time to pubic hair stage>1**		**Risk of earlier time to menarche**	
	**Male**	**Female**	**Male**	**Female**	**Male**	**Female**	**Male**	**Female**
**Faster velocity of weight gain**								
. Early infancy	Earlier APHV	**X**	**X**	**X**	**X**	**X**	NA	**X**
. Late infancy	Earlier APHV	**X**	**X**	**X**	**X**	**X**	NA	**X**
. Early childhood	Earlier APHV	Earlier APHV	**X**	**X**	**X**	Increased risk	NA	**X**
**Faster velocity of length/height gain**								
. Early infancy	Earlier APHV	**X**	**X**	**X**	**X**	**X**	NA	**X**
. Late infancy	Earlier APHV	**X**	**X**	**X**	**X**	**X**	NA	**X**
. Early childhood	Earlier APHV	Later APHV	**X**	**X**	**X**	**X**	NA	**X**
**Faster velocity of BMI gain**								
. Early infancy	Earlier APHV	**X**	**X**	**X**	**X**	**X**	NA	**X**
. Late infancy	Earlier APHV	**X**	**X**	**X**	**X**	**X**	NA	Increased risk
. Early childhood	Earlier APHV	Earlier APHV	**X**	**X**	**X**	Increased risk	NA	**X**

X = no significant association; NA = not applicable.

© 2022 Aris IM, et al. *JAMA Netw Open.*

eFigure. Directed Acyclic Graph Representing the Possible Relationship Among Baseline Covariates, Child Growth, and Pubertal Outcomes

The variable *L* represents the baseline covariates included in this study (i.e., child race, Hispanic ethnicity, gestational age at delivery, birth year, maternal age at delivery, maternal education level, and household income during pregnancy, prenatal cigarette smoking, parity, pre-pregnancy body mass index, gestational diabetes, gestational hypertension, preeclampsia, total gestational weight gain, and mode of delivery). The variable *U* represents common causes of child growth and pubertal development that were not accounted for in our study (e.g., child diet and/or physical activity).

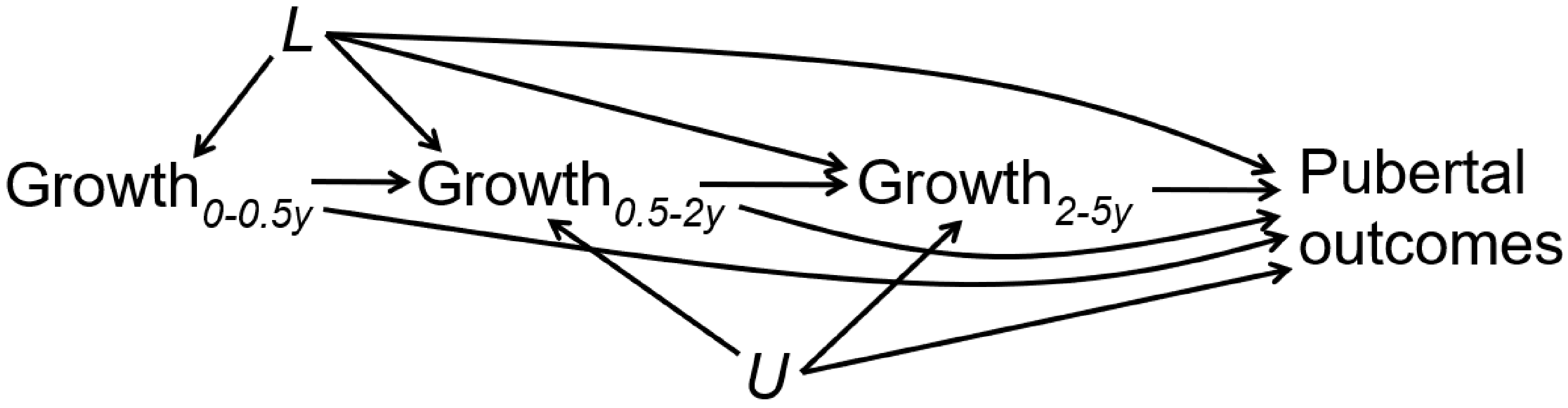

© 2022 Aris IM, et al. *JAMA Netw Open.*

eMethods. Procedure for Estimating Growth Velocities in the First 5 Years of Life

We estimated individual-level rates of weight (kg/year), length/height (cm/year) and body mass index (BMI; kg/m^2/year) gain across early infancy (0–0.5 years), late infancy (0.5–2 years), and early childhood (2–5 years) using linear spline mixed-effects models. Briefly, this approach models the growth trajectory as a series of connected lines (i.e., a piecewise model) that allows for different linear slopes from 0–0.5 years, 0.5–2 years, and 2–5 years, with these slopes varying between individuals. These models are fitted with maximum-likelihood estimation,[1] a method of estimating the parameters of an assumed probability distribution, given the observed data. Specifically, this method allows us to obtain parameter estimates (i.e., population average velocities, as well as deviations from the average velocity for each child, of weight, length/height, or BMI gain in early infancy, late infancy, and early childhood) even in the presence of missing data. It does not impute any data, but rather, uses each individual's available data to compute the maximum likelihood. Thus, these models enable estimation of growth trajectories in children even if they only had one measure of anthropometry in the first 5 years of life, with the caveat that children with a greater number of missing measurements would have growth trajectories that are closer to the population average. Such models also allow for changes in scale and variance of weight, length/height, and BMI over time.[2] The model is given by:

$$Y_{ij} = \beta_0 + \mu_{0i} + (\beta_1 + \mu_{1i}) s_{1i} + (\beta_2 + \mu_{2i}) s_{2i} + (\beta_3 + \mu_{3i}) s_{3i} + e_{ij}$$

where β_0 is the population average intercept at birth and β_1, β_2, and β_3 are the population average velocities of weight, length/height, or BMI gain in early infancy (s_1), late infancy (s_2), and early childhood (s_3), respectively. μ_{0i} is the deviation from the average intercept for child i, and μ_{1i}, μ_{2i}, and μ_{3i} are the deviations for child i from the average velocities of weight, length/height or BMI gain in early infancy, late infancy, and early childhood, respectively (i.e., the individual-level random effects). e_{ij} is the deviation from the predicted size for child i at age j. We used an unstructured covariance matrix so that each random effect would be correlated with all other random effects.

From these models, we obtained individual-level estimates of birth size and velocities of weight, length/height, and BMI gain across the three age periods for each child. Previous studies have shown that comparisons of absolute rates of weight, length/height, or BMI gain in different periods of life will be affected by underlying differences in growth rates, as well as duration, at different ages and may bias inferences about their relative importance for later outcomes.[3,4] To allow for direct comparison of growth velocities across different age periods, we standardized these growth velocities as z-scores with a mean of 0 and SD of 1. We calculated these standardized values using internally derived sex-specific means and standard deviations.

eReferences.

1. Verbeke G. Linear Mixed Models for Longitudinal Data. In: Linear Mixed Models in Practice, Springer, New York. 1997;126:63-153.
2. Goldstein H. Efficient statistical modelling of longitudinal data. Ann Hum Biol. 1986;13(2):129-141.
3. Zhang X, Martin RM, Oken E, Aris IM, Yang S, Kramer MS. Growth During Infancy and Early Childhood and Its Association With Metabolic Risk Biomarkers at 11.5 Years of Age. American journal of epidemiology. 2020;189(4):286-293.
4. Zhang X, Tilling K, Martin RM, et al. Analysis of 'sensitive' periods of fetal and child growth. Int J Epidemiol. 2019;48(1):116-123.

© 2022 Aris IM, et al. *JAMA Netw Open.*
