# Supplementary material for: Analysis of Early-Life Growth and Age at Pubertal Onset in US Children
Source: JAMA Netw Open. 2022 Feb 4;5(2):e2146873. doi: 10.1001/jamanetworkopen.2021.46873 (PMC8817204; doi:10.1001/jamanetworkopen.2021.46873)
Supplement: Supplement 2. — Nonauthor Collaborators [file jamanetwopen-e2146873-s002.pdf]

Supplemental Online Content: Nonauthor Collaborators

*Indicates required information. Only first name, last name, and suffix will appear in PubMed.

*Group Name(s): Program Collaborators for Environmental Influences on Child Health Outcomes							
***First Name and Middle Initial(s)**	***Last Name**	***Suffix (eg, Jr, III)**	Academic Degrees	Institution	Location (city, state/province, country)	Role or Contribution, eg, chair, principal investigator	Group (if more than 1 Group listed in the byline) and/or Subgroup (eg, Steering Committee)
Akram	Alshawabkeh		PhD	Northeastern University	Boston, MA, USA	Principal Investigator	
Judy	Aschner		MD	Albert Einstein College of Medicine	Bronx, NY, USA	Principal Investigator	
Clancy	Blair		PhD	New York University	New York, NY, USA	Principal Investigator	
Carlos	Camargo	Jr	MD	Massachusetts General Hospital	Boston, MA, USA	Principal Investigator	
Sean	Deoni		PhD	Memorial Hospital of Rhode Island	Pawtucket, RI, USA	Principal Investigator	
Cristiane	Duarte		PhD	Columbia University	New York, NY, USA	Principal Investigator	
Anne	Dunlop		MD	Emory University	Atlanta, GA, USA	Principal Investigator	
Amy	Elliott		PhD	University of Michigan	Ann Arbor, MI, USA	Principal Investigator	
Assiamira	Ferrara		MD	Kaiser Permanente	Oakland, CA, USA	Principal Investigator	
James	Gern		MD	University of Wisconsin- Madison	Madison, WI, USA	Principal Investigator	
Carrie	Breton		ScD	University of Southern California	Los Angeles, CA, USA	Principal Investigator	
Irva	Hertz-Picciotto		PhD	University of California, Davis	Davis, CA, USA	Principal Investigator	
Alison	Hipwell		PhD	University of Pittsburgh	Pittsburgh, PA, USA	Principal Investigator	
Margaret	Karagas		PhD	Dartmouth College	Hanover, NH, USA	Principal Investigator	
Catherine	Karr		MD, PhD	University of Washington	Seattle, WA, USA	Principal Investigator	

Supplemental Online Content: Nonauthor Collaborators

*Indicates required information. Only first name, last name, and suffix will appear in PubMed.

***First Name and Middle Initial(s)**	***Last Name**	***Suffix (eg, Jr, III)**	Academic Degrees	Institution	Location (city, state/province, country)	Role or Contribution, eg, chair, principal investigator	Group (if more than 1 Group listed in the byline) and/or Subgroup (eg, Steering Committee)
Barry	Lester		PhD	Women & Infants Hospital of Rhode Island	Providence, RI, USA	Principal Investigator	
Leslie	Leve		PhD	University of Oregon	Eugene, OR, USA	Principal Investigator	
Johnnye	Lewis		PhD	University of New Mexico	Albuquerque, NM, USA	Principal Investigator	
Scott	Weiss		MD	Brigham and Women's Hospital	Boston, MA, USA	Principal Investigator	
Cynthia	McEvoy		MD	Oregon Health & Science University	Portland, OR, USA	Principal Investigator	
Craig	Newschaffer		PhD	Drexel University	Philadelphia, PA, USA	Principal Investigator	
Thomas	O'Connor		PhD	University of Rochester	Rochester, NY, USA	Principal Investigator	
Jean	Kerver		PhD	Michigan State University	Lansing, MI, USA	Principal Investigator	
Julie	Herbstman		PhD	Columbia University	New York, NY, USA	Principal Investigator	
Susan	Schantz		PhD	University of Illinois	Urbana, IL, USA	Principal Investigator	
Joseph	Stanford		MD	University of Utah	Salt Lake City, UT, USA	Principal Investigator	
Leonardo	Trasande		MD	New York University	New York, NY, USA	Principal Investigator	
Rosalind	Wright		MD	Icahn School of Medicine at Mount Sinai	New York, NY, USA	Principal Investigator	
